# Spine Trauma Resource Priorities in Sub-Saharan Africa: A Delphi Approach

**DOI:** 10.1177/21925682251383510

**Published:** 2025-10-10

**Authors:** Charlotte F. Wahle, Chellandra Samuels, Shravya Kakulamarri, Babapelumi Adejuyigbe, Trisha Tee, Francisco Gomez Alvarado, Saam Morshed, Ashraf N. El Naga, David W. Shearer, David Gendelberg, East Africa Spine Trauma Network, Africa Spine Trauma Delphi Study Group

**Affiliations:** 1 Institute for Global Orthopaedics and Traumatology, 8785 UCSF, San Francisco, CA, USA; 2 158263 Herbert Wertheim College of Medicine, Miami, FL, USA; 3 Department of Orthopedic Surgery, 8783 University of California, San Francisco, CA, USA

**Keywords:** spinal navigation, orthopaedic, deformity, cervical, lumbar, thoracic, fusion, trauma, radiology

## Abstract

**Study Design:**

Delphi study.

**Objective:**

Optimal spine trauma care requires extensive resource availability and training. In LMICs, where resources and training are scarcer, spine trauma represents a major source of disability and public health burden. This study aimed to evaluate the highest priority resource needs for spine surgeons in sub-Saharan Africa in order to provide safe, high-quality spine trauma care.

**Methods:**

This study utilized a Delphi methodology with three electronic surveys. Participants were identified through the Continental Association of African Neurosurgical Societies (CAANS) and College of Surgeons of East, Central, and Southern Africa (COSECSA) networks. Response collection lasted from August 2024 to May 2025. The initial survey was an open-ended collection of all spine trauma resource priorities. In each subsequent round, participants rated topics on a Likert scale from 1 (most important) to 9 (least important). Consensus was defined as topics ranked 1, 2 or 3 (highly important) by >70% of participants.

**Results:**

Invitations were sent to 75 potential participants, with 36 participating in round one. Thirty-four participants (94.4%) and thirty-five (97.2%) completed the second and third consensus rounds respectively. Fifty-one items reached consensus. The most highly rated items included braces/c-collars, spine training/education of emergency room physicians and surgeons, and access to implants. Items not reaching consensus included microscopes, endoscopes, navigation and specialized auxiliary personnel.

**Conclusion:**

Given the high burden of traumatic spine injury and resource needs in these regions, efforts should be focused on providing essential resources related to timely stabilization of the spine over advanced tools and technologies.

## Introduction

Traumatic spine injury is a major global cause of both disability and mortality.^1,2^ These injuries are often highly time-sensitive and resource-intensive, posing significant challenges for optimal treatment.^3^ The burden of spine trauma falls disproportionately on low- and middle-income countries (LMICs), where resources are limited.^1,4^ In Africa, the estimated incidence of spine trauma is approximately 13.6 cases per 100,000 individuals, compared to 10.5 cases per 100,000 at a global scale, which is an important and likely underestimated disparity.^1^ There is also notable intracontinental variation, with some countries and regions reporting considerably higher burdens than others.^5-7^ These differences are likely multifactorial. However varying levels of resources both across and within these countries uniquely affect the management of spine trauma patients.

Given the high risk of morbidity and mortality, timely and appropriate care across pre-hospital, emergency department, surgery, intensive care, and post-operative care settings is critical to optimal outcomes in traumatic spinal injury. However, limited access and insufficient training in under-resourced settings negatively impacts care delivery.^8^ A lack of both material and human resources is a major barrier to the effective application of spine trauma management guidelines.^4^ In response, recent efforts have focused on developing adaptable management protocols that can accommodate variations in local resources and training.^4^ Nevertheless, certain fundamental resources remain essential for delivering optimal spine trauma care, even with a flexible management protocol.

To support guideline development and advocate for the availability of essential resources, it is first necessary to understand current resource capacity across different countries and regions. Thus, the aim of this study was to identify the highest priority resource needs, as determined by an expert panel of local spine surgeons in sub-Saharan Africa, to inform efforts directed at providing safe, high-quality spine trauma care.

## Methods

The study followed a Delphi survey approach. The survey was conducted between the months of August 2024 and May 2025. The survey was administered in English and the data was stored via a REDCap server.

### Expert Acquisition

To identify experts in the field, a participant interest form was circulated via WhatsApp and email, with recipients encouraged to share the link with colleagues. The interest form was also distributed at relevant local scientific meetings, including the Continental Association of African Neurosurgical Societies (CAANS) and the College of Surgeons of East, Central, and Southern Africa (COSECSA). Exclusion criteria consisted of those who had not completed residency, did not perform spine surgery, or did not practice in Sub-Saharan Africa.

### Delphi Round 1

In the first round, surgeons’ demographic information was collected and participants were given an open answer box to list all resources they considered essential for optimal spine trauma care. Demographic variables included age, sex, country, years in practice, practice setting, academic affiliation, and medical specialty (neurosurgery or orthopaedic surgery). Participants could list an unlimited number of resources that they deemed critical for optimal care in spine trauma surgery. The survey responses were subsequently grouped into several categories: Pre-Hospital, In-Hospital - Personnel, In-Hospital - Supplies/Equipment, Infrastructure, Post-Operative Care/Rehabilitation, Training/Education, Research, and Miscellaneous.

### Delphi Round 2

A list of the sixty-three topics identified from Delphi Round 1 was compiled in an electronic survey and distributed to the participating surgeons. Participants were asked to review and rate each topic on its perceived value to optimal care in spine trauma surgery using a 9-point Likert scale with 1-3 being “most important”, 4-7 being “moderately important”, and 7-9 being “least important”. Round 2 responses were then collected and analyzed using STATA (StataCorp, College Station, TX). A histogram was generated for each item to show the distribution of participant responses.

### Delphi Round 3

During Delphi Round 3, a third electronic survey was administered to all participants. This survey included the histograms showing the mean ratings and distribution for each topic in Round 2. Using the same Likert scale as Round 2, participants were asked to re-rate each item based on their perception of its importance with the added context of the ratings that others in the group had given the item. Statistical analysis was again performed using STATA. Consensus on topics was defined as those rated 1, 2 or 3 (highly important) by ≥70% of the sampled group.

## Results

### Demographics and First Round of the Survey

Initial invitations were electronically sent to 75 potential participants across the CAANS and COSECSA networks. Of the 75 potential participants, a total of 36 experts agreed to participate and responded to the first round of the survey (Table 1). The vast majority (94.4%) were male. The expert panel included both neurosurgeons and orthopaedic surgeons, with a higher representation of neurosurgeons (77.8%) compared to orthopaedic surgeons (22.2%). The group represented twelve different African countries, with the largest numbers coming from Ethiopia (10, 27.8%), Nigeria (9, 25.0%), Ghana (3, 8.3%) and Kenya (3, 8.3%). All participants reported some affiliation with an academic institution. A majority (91.7%) reported spending at least some of their practice taking care of publicly insured patients, while a third (33.3%) take care of privately insured patients. Levels of experience also varied, with twenty-one respondents (58.3%) between 0 to 5 years in practice, 27.8% between 6-10 years in practice, and 13.9% between 11-20 years in practice. Following initial survey completion, participants submitted 63 unique items as resource priorities for spine trauma care.

**Table 1.** Respondent Demographics

Category total	N	%
Country		
Benin	1	2.8
Burkina Faso	1	2.8
Côte d'Ivoire	2	5.6
Ethiopia	10	27.8
Gambia	1	2.8
Ghana	3	8.3
Kenya	3	8.3
Mali	1	2.8
Malawi	2	5.6
Nigeria	9	25.0
Somalia	1	2.8
Tanzania	2	5.6
Sex		
Male	34	94.4
Female	2	5.6
Years in practice		
0-5 years	21	58.3
6-10 years	10	27.8
11-15 years	3	8.3
16-20 years	2	5.6
Practice setting (can select multiple)		
Private clinic/hospital	12	33.3
Public clinic/hospital	33	91.7
Academic affiliation		
Yes	36	100
No	0	0
Practice type		
Neurosurgery	28	77.8
Orthopaedic Surgery	8	22.2

### Second and Third Rounds of the Survey

A total of thirty-four participants (94.4%) completed the second round of the survey. Each resource item was rated using a 9-point Likert scale, with 1 indicating “most important” and 9 indicating “least important”. The mean score for each resource is reported in Table 2. Thirty-five (97.2%) completed the final round of the survey.

**Table 2.** Overall Ratings of Spine Trauma Resources and the Percentage of Consensus Among Participants

Topics (Round 1)	Round 2 Mean^a^	Round 3 Consensus^b^
Pre-Hospital		
Ambulance Services	1.71	88.5%
Emergency Medical Technicians / Paramedics	2.06	94.3%
Training/protocol for pre-hospital care	1.88	94.3%
Spine boards for pre-hospital care	2.0	82.9%
Braces/c-collars for pre-hospital care	1.62	97.1%
In-Hospital – Personnel		
Spine-trained surgeon (orthopaedic or neuro)	1.38	97.1%
Operating Room nurses	2.03	91.4%
Physical Therapist	2.21	91.4%
Physiatrist - Physical Medicine and Rehabilitation	2.82	80.0%
In-Hospital – Supplies/Equipment		
X-ray	2.65	74.3%
CT	1.88	91.4%
MRI	1.68	82.9%
Fluoroscopy	2.26	91.4%
Implants – anterior cervical system (interbody and plate)	1.68	97.1%
Implants – corpectomy system	2.24	85.7%
Implants – posterior instrumentation (rods and screws)	1.53	97.1%
Intraoperative neuromonitoring	3.15	91.4%
Dressings (bandages, gauze, other)	3.24	77.1%
Antibiotics	3.03	77.1%
Infrastructure		
Dedicated trauma wards	2.44	74.3%
Intensive care unit - general	2.09	85.7%
Intensive care unit – trauma/surgery	2.26	88.6%
Intensive care unit – neurology/neurosurgery	2.24	94.3%
Dedicated trauma ORs	2.0	88.6%
Dedicated spine ORs	2.44	74.3%
Rehabilitation facilities	2.06	82.9%
Post-operative Care/Rehabilitation		
Rehab aids (gym, PT stairs, shockwaves, other)	2.74	77.1%
Ambulatory aids (crutches, walker, wheelchair)	2.36	91.4%
Bean bag/air mattress (for pressure ulcers)	2.73	71.4%
Physical Therapy	1.79	88.6%
Occupational Therapy	2.76	74.3%
Physical Medicine and Rehabilitation Services	2.74	74.3%
ICU care, monitoring and evaluation protocols	1.74	88.6%
Skilled nursing (wound care, home care)	2.44	88.6%
Training/Education		
Training (emergency room – staff/nursing)	2.06	94.3%
Training (emergency room – physicians)	2.09	97.1%
Physical Therapy/Occupational Therapy (on spine rehabilitation protocols)	2.35	91.4%
Training (operating room) - staff/nursing	2.09	91.4%
Training (operating room) - anesthesia	2.18	88.6%
Training (operating room) – orthopaedic surgeon/neuro	1.79	94.3%
Training (operating room) – trauma surgeon	2.15	85.7%
Training – Spine fellowship	1.68	94.3%
Education for patients on rehabilitation and follow-up	2.45	91.4%
Research		
Spine trauma registry/Clinical outcomes research	1.65	94.3%
Spine research funding	2.0	94.3%
Spine research education (experimental design)	1.88	88.6%
Spine research support (research coordinators)	2.24	91.4%
Spine research collaborations (within country)	1.79	88.6%
Spine research collaborations (out of country)	2.15	91.4%
Miscellaneous		
Health Insurance	2.21	85.7%
Advocacy at the policy level	1.82	94.3%

^a^Likert scale with 1 being most important and 9 being least important.

^b^Consensus was considered >70% 1, 2 or 3.

In total, fifty-one items reached consensus, meaning they received >70% agreement of one, two or three on the Likert scale indicating a high degree of importance (Table 2). The most highly rated items included braces/c-collars (97.1%), training/education of emergency room physicians on spine trauma management (97.1%), spine-trained surgeons (97.1%), and access to spine implants — including anterior cervical systems (97.1%) and posterior instrumentation (97.1%). Additionally, all items in the Research and Post-operative Care/Rehabilitation categories reached consensus.

Twelve items did not reach consensus. The category with the greatest number of items that did not reach consensus was in In-Hospital Supplies/Equipment (Table 3). Several of the items in this category included highly specialized spine items, such as neuro-radiologists, neuro-anesthesiologists, spine tables, endoscopes and spine navigation systems, were rated as lower priority on the Likert scale and did not meet the consensus threshold.

**Table 3.** Spine Trauma Resources That Did Not Reach Consensus

Topics (Round 1)	Round 2 Mean^a^	Round 3 Consensus^b^
Pre-Hospital		
Simulation training (realistic practice of clinical scenarios)	2.68	65.7%
Steroids availability and education	5.15	34.3%
In-Hospital – Personnel		
Neuro-Anesthesiologist	2.47	68.6%
Neuro-Radiologist	3.15	57.1%
In-Hospital – Supplies/Equipment		
Spine table	2.62	65.7%
Microscope	2.68	54.3%
Endoscope	3.68	37.1%
Navigation	4.12	31.4%
Analgesia	2.59	65.7%
Infrastructure		
Dedicated trauma treatment rooms (shock rooms)	2.82	62.9%
Training – Minimally invasive surgery (MIS)	2.68	65.6%
Education for patients on financial support	3.06	68.6%

^a^Likert scale with 1 being most important and 9 being least important.

^b^Consensus was considered >70% 1, 2 or 3.

## Discussion

Optimal spine trauma care often depends on both specialized equipment and extensive training. While efforts are being made to help ensure that low resource areas can access the essential tools needed to provide this life-saving care, it remains difficult to triage which tools and capacities are needed most urgently due to the complex, multidisciplinary nature of spine trauma care. This study aimed to identify essential resource priorities by surveying a panel of experts across sub-Saharan Africa. Thirty-six experts from twelve countries participated in the survey. By the conclusion of the study, a total of fifty-one items reached consensus on a 9-point Likert scale from least essential to most essential. Among the highest needs were elements of pre-hospital care, spine-specific trauma education and training, and access to affordable implants. In contrast, items that failed to reach consensus generally involved high cost or highly specialized tools, such as endoscopes, microscopes, and neuro-specific anesthesiologists or radiologists.

### Importance of Prehospital Care

The findings underscore the critical role of pre-hospital management in optimizing spine trauma outcomes. Braces and cervical collars were among the most highly rated resources, emphasizing the importance of spinal immobilization both at the scene of injury and in transit. Similarly, other pre-hospital resources, such as ambulance transport, emergency medical personnel, and structured triage protocols were also recognized as essential for optimal spine trauma response. The strong prioritization of pre-hospital care is supported by existing literature, highlighting the significant impact of early and appropriate intervention on patient outcomes. The ability of first-line providers to promptly recognize and manage spinal injuries in the field plays a crucial role in shaping long-term neurological recovery.^9,10^ If critical early decisions are delayed, the chances of favorable outcomes can be catastrophic and irreversible.^10^ In a 2006 study in Tanzania, Härtl et al^11^ reported a significantly higher mortality rate among patients undergoing indirect transfers, illustrating the importance of streamlined referral and transport systems. Moreover, early, protocol-driven intervention has also been shown to improve surgical outcomes, particularly in patients with acute neuro deficit.^12^ Unfortunately, many LMICs lack the infrastructure to systematically pre-hospital care. In a more recent study by Magogo et al^13^ reported delays between injury and hospital admission is exacerbated in regions where neurosurgical centers are sparse, as in Tanzania. Similarly, in the Democratic Republic of Congo, Beltchika et al^14^ describe how the absence of organized emergency medical services, limited training opportunities, and shortages of surgical implants and imaging equipment all hinder timely and effective spine trauma care. Taken together, these findings and the present study support the pivotal role of pre-hospital management in building a foundation for effective spine trauma care.

### Access to Spine Surgery Fellowship/Training

Access to spine-trained surgeons also emerged as a top priority for optimal spine trauma care, reinforcing the need for dedicated spine fellowships and education infrastructure. While spine training is often incorporated in orthopaedic and neurosurgical residencies, its depth and quality is greatly influenced by the program’s spine caseload and mentorship opportunities.^15^ Consequently, orthopedic and neurosurgeons without formal spine fellowships may have limited confidence and experience managing complex spine trauma without completing a spine fellowship.^15^ In several African nations, including Nigeria, the growing burden of spine trauma and subsequent surgical demand has outpaced training capacity.^16,17^ Critical educational barriers exist in these areas, including a shortage of qualified trainers, institutional resources and a lack of formal fellowship opportunities. Through expanding access to spine-specific training through collaborative partnerships, visiting observerships, and formal education programs such as fellowships, the global spine community can exchange invaluable knowledge and insights — strengthening capacity for high-quality global spine trauma care.

### Access to Implants

Two of the most endorsed resources were spine surgery implant systems, including both anterior cervical systems and posterior instrumentation. Hardware is central to most spine trauma surgeries, playing a critical role in stabilization, surgical planning and long-term outcomes.^18-20^ In many African countries, patients must purchase the implants out of pocket before they can undergo surgery.^12,14,21,22^ For uninsured patients and those living in poverty, this requirement can be insurmountable – leading to days or week-long delays to definitive treatment or catastrophic expenditure.^12,14,17^ At Muhimbili Orthopaedic Institute in Tanzania, Leidinger et al^12^ found that the mean time to surgery for spine trauma cases was 33 days, with financial barriers being the primary driver of delay. More recent data from the Democratic Republic of the Congo shows even longer delays, with a mean time to surgery of 62 days. Interestingly, this paper also found that only 63% of patients who underwent instrumentation received the appropriate implants for their injury, while the remaining 27% received “improvised elements”.^14^ These delays have direct implications for patient well-being — leading to worse neurologic recovery and lower likelihood of return to function.^12,17,22^ The present study’s consensus findings as well as these reports make clear that implant availability is clearly an essential resource for delivering effective spine trauma care.

### Low Consensus Resources

Resources that yielded lower consensus among the group of experts included both physical equipment and educational training supporting specialized or advanced surgical techniques, such as endoscopic/minimally invasive surgery or neuro-specialized anesthesia and radiology teams. These discrepancies align with previous research showing significant differences in resource prioritization across income groups in musculoskeletal trauma.^23,24^ Surgeons from HICs often rank specialized personnel and more advanced technologies more highly than those from LMICs and UMICs.^24^ By contrast, some individuals in LMICs may perceive such technologies as “extravagant” when so many basic needs are unmet. Given that the safety and cost-effectiveness of traditional open spine surgery has been more robustly researched, especially in LMICs, the experts in the study may have prioritized only those tools essential for acute stabilization. On the other hand, many of these advanced surgical techniques have been associated with reduced infection rates, morbidity, and destabilization risk across varying levels of surgical complexity, which may explain why some experts still ranked them as high priority, contributing to the lack of consensus.^25-27^ In fact, a 2023 study by Tayal et al^28^ demonstrated surgeons’ support for broader integration of endoscopy use and training in LMICs. Thus, while these technologies may ultimately reduce resource burden by enabling faster surgeries with fewer complications, their high upfront cost remains a major barrier to prioritization until basic needs are met.^27^

### Limitations

This study has several limitations. While participants identified a broad group of resources, it is possible that some, particularly very basic resources, may have been overlooked. Additionally, several aspects of this study may have limited the generalizability of the results, including its small sample size, variation in participants’ years of experience, limited geographic diversity, and a primarily male sample population. Further, the study only included spine surgeons, excluding valuable perspectives from other non-surgeon stakeholders such as emergency physicians, nurses, rehabilitation providers, or patients. There is also potential for selection bias. Experts were recruited through two professional societies, which may have skewed the sample toward individuals with academic affiliations. Finally, the study was conducted in English, which may have limited participation from non-English-speaking respondents.

## Conclusion

Following a comprehensive Delphi survey of thirty-six spine trauma surgery experts across sub-Saharan Africa, this study reached consensus on the highest priority resource needs for spine trauma care. The most highly rated items primarily related to pre-operative and intraoperative spine stabilization, while more specialized tools and technologies were consistently rated as lower priority. Given the high burden of disease and limited resources in these regions, efforts should prioritize the provision of essential, high-impact resources before considering the implementation of more advanced or specialized interventions.

## Data Availability

The survey data is available from the corresponding author upon request.*
